# A Tale of Two Congenital Lesions: A Case Report of Congenital Diaphragmatic Hernia and Congenital Heart Disease Managed by Successful Surgical Outcome With Review of the Literature (Bhende-Pathak Hernia)

**DOI:** 10.7759/cureus.75238

**Published:** 2024-12-06

**Authors:** Vishal V Bhende, Mahesh H Bhatt, Viral B Patel, Rahul Tandon, Mathangi Krishnakumar

**Affiliations:** 1 Pediatric Cardiac Surgery, Bhanubhai and Madhuben Patel Cardiac Centre, Shree Krishna Hospital, Bhaikaka University, Karamsad, IND; 2 Pediatric Interventional Cardiology, Bhanubhai and Madhuben Patel Cardiac Centre, Shree Krishna Hospital, Bhaikaka University, Karamsad, IND; 3 Radiodiagnosis and Imaging, Pramukhswami Medical College and Shree Krishna Hospital, Bhaikaka University, Karamsad, IND; 4 Pediatrics, Pramukhswami Medical College and Shree Krishna Hospital, Bhaikaka University, Karamsad, IND; 5 Anaesthesiology, St. John’s Medical College Hospital, Bengaluru, IND

**Keywords:** • bhende-pathak hernia, bochdalek's hernia, congenital diaphragmatic hernia, congenital heart disease, diaphragm, ileo-caecal appendix, mesh repair, patent ductus arteriosus, pulmonary hypoplasia, surgical ligation of patent ductus arteriosus

## Abstract

Congenital diaphragmatic hernia (CDH) is a diaphragmatic defect that is usually situated on the left side in the posterolateral region, named a Bochdalek hernia (BH), which allows abdominal organs to herniate into the thoracic cavity. BH is a prevalently observed birth anomaly in infants but is rare in adults. Right-sided BH that involves the colon is exceptionally rare, and no prior cases have described ileocecal appendix involvement. Here, we present a case of a preschooler with a right-sided BH and patent ductus arteriosus (PDA), requiring distinct surgical approaches: left open thoracotomy for PDA ligation and right open thoracotomy for CDH repair. Surgical intervention is associated with reduced morbidity and mortality, favorable long-term outcomes, and a low recurrence rate, irrespective of the selected approach. Reinforcement through suture repair with mesh application, as well as PDA ligation, reinforced with metallic clips as the preferred surgical operation in this case. To the best of our knowledge, this is the first reported instance of a pediatric patient with right-sided BH that involves the appendix, alongside concurrent congenital heart disease (CHD). We propose the term Bhende-Pathak hernia for this pediatric variant.

## Introduction

Congenital diaphragmatic hernia (CDH) is a rare but serious congenital defect occurring in 2.5 to 3.8 per 10,000 births [1]. CDH is characterized by a structural defect in the diaphragm that allows abdominal organs to protrude into the thoracic cavity, resulting in pulmonary hypoplasia, abnormal pulmonary vascular development, and altered vasoreactivity. Pulmonary hypertension (PH) frequently complicates CDH and is strongly associated with increased morbidity and mortality in affected individuals [1-4]. Early detection and management of PH are essential for improving outcomes in CDH, and PH severity is a critical prognostic factor in CDH care [5-7].

Bochdalek hernia (BH), the most common type of CDH, occurs in approximately one in 2,000 to one in 5,000 live births [8,9]. Most BH cases are identified prenatally or in the neonatal period, with only 5% diagnosed beyond the neonatal stage [8,10,11]. BH is exceptionally rare in adults, accounting for just 0.17% to 6% of all diaphragmatic hernias [8,12]. In a 1984 study by Gale involving 940 consecutive computed tomography (CT) scans, the prevalence of BH was reported at 6% [13]. Right-sided BH involving the colon is particularly uncommon, with only 12 cases reported globally [14]. By 2002, only seven cases of symptomatic right-sided BH had been documented in the literature [8].

In CDH, underdevelopment of the affected lung can lead to elevated pulmonary vascular resistance, PH, and, potentially, right heart failure [15]. Mortality rates vary based on individual patient factors but range from 10% to 35% [16]. In patients with CDH, a patent ductus arteriosus (PDA) can serve as a compensatory mechanism for elevated pulmonary pressures by allowing pulmonary-to-systemic shunting, which helps relieve right heart strain. As the disease progresses and PH decreases, however, the flow through the PDA may reverse, increasing left heart strain and necessitating closure of the PDA to prevent complications from a significant left-to-right shunt [17].

We report on a four-year-old girl with a congenital right-sided BH involving the appendix as herniated content, accompanied by concurrent CDH. This unique case, termed the Bhende-Pathak hernia, represents a pediatric variant of BH.

## Case presentation

The Institutional Ethics Committee (IEC-2) of the H.M. Patel Center for Medical Care and Education in Anand, Gujarat (which is affiliated to Bhaikaka University, Karamsad), approved this study (Approval No. IEC/BU/2024/Cr.64/339/2024, dated October 14, 2024). The patient’s parents provided their consent to use the medical data of their child.

A four-year-old girl, born full-term at 2.5 kg via normal vaginal delivery, was received at the pediatric outpatient clinic of the Department of Pediatrics at Shree Krishna Hospital, Bhaikaka University, Karamsad, with the recent-onset of abdominal pain. The pain was localized to the right hypochondriac and epigastric regions, was dull and aching, did not radiate, and had no association with food intake, aggravating or relieving factors. There was no history of chest trauma or hemoptysis. At 15 days of age, she had been evaluated by a local practitioner for breastfeeding difficulties, and two-dimensional echocardiography revealed a moderate PDA with left-to-right shunting and mild pulmonary arterial hypertension.

**Figure 1:**
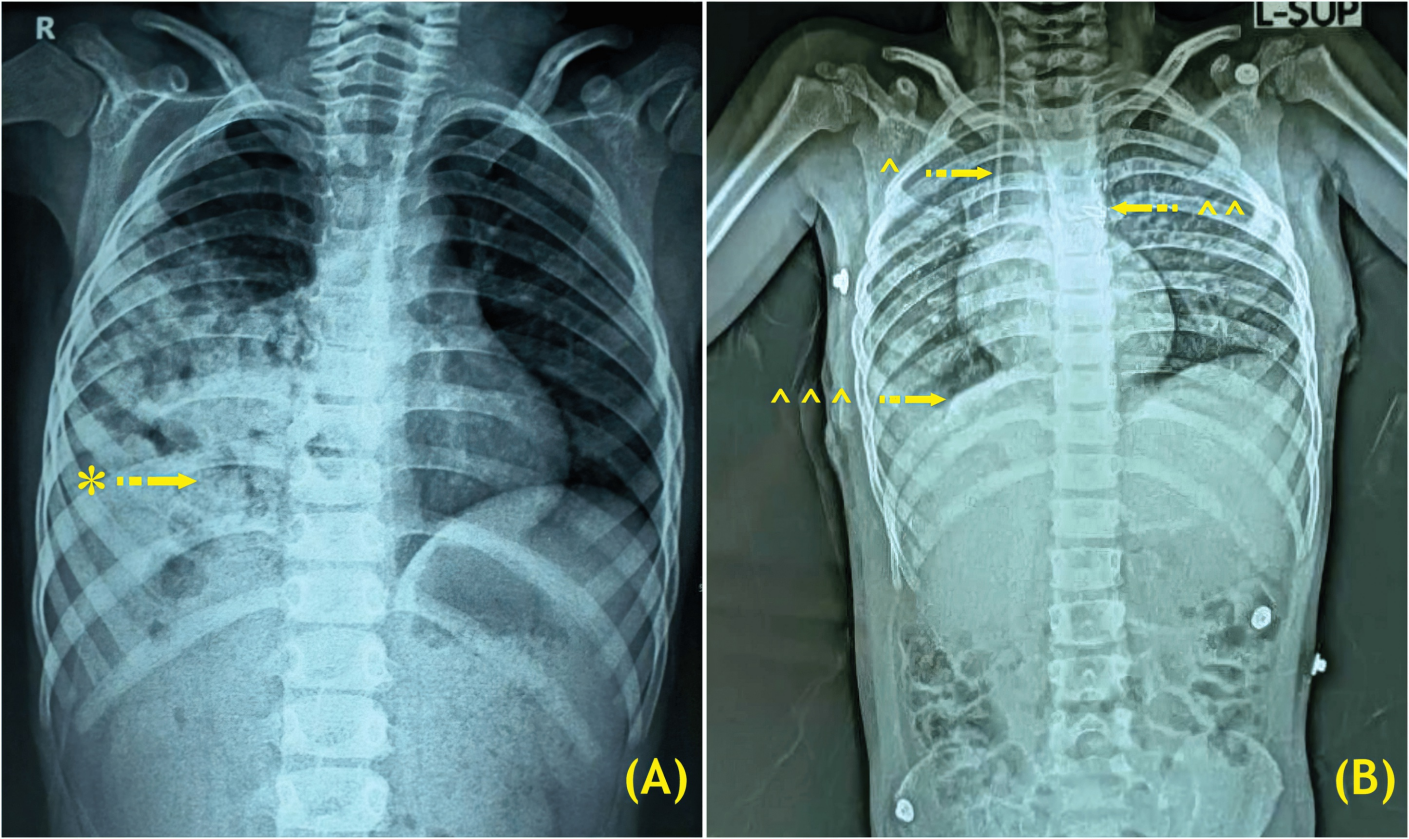
Chest X-ray showing right-sided Bochdalek hernia (BH) and patent ductus arteriosus (PDA) preoperatively (A) and postoperatively (B). * Right-sided large congenital diaphragmatic hernia (CDH) (A); ^ Central venous pressure (CVP) line; ^^ Double ligation of PDA with reinforcement of metallic clip; ^^^ Right CDH primary repair with Parietex™ Optimized Composite Mesh (Covidien) (B). BH, Bochdalek hernia ; PDA, patent ductus arteriosus; CDH, congenital diaphragmatic hernia; CVP, central venous pressure. (Image credits: Dr. Vishal V. Bhende)

Clinical examination revealed reduced breath sounds in the infra-mammary area and heart sounds on the right side of the thorax. Blood tests indicated a complete blood count within the reference range. A posteroanterior chest X-ray suggested right-sided CDH (Figure 1).

**Figure 2:**
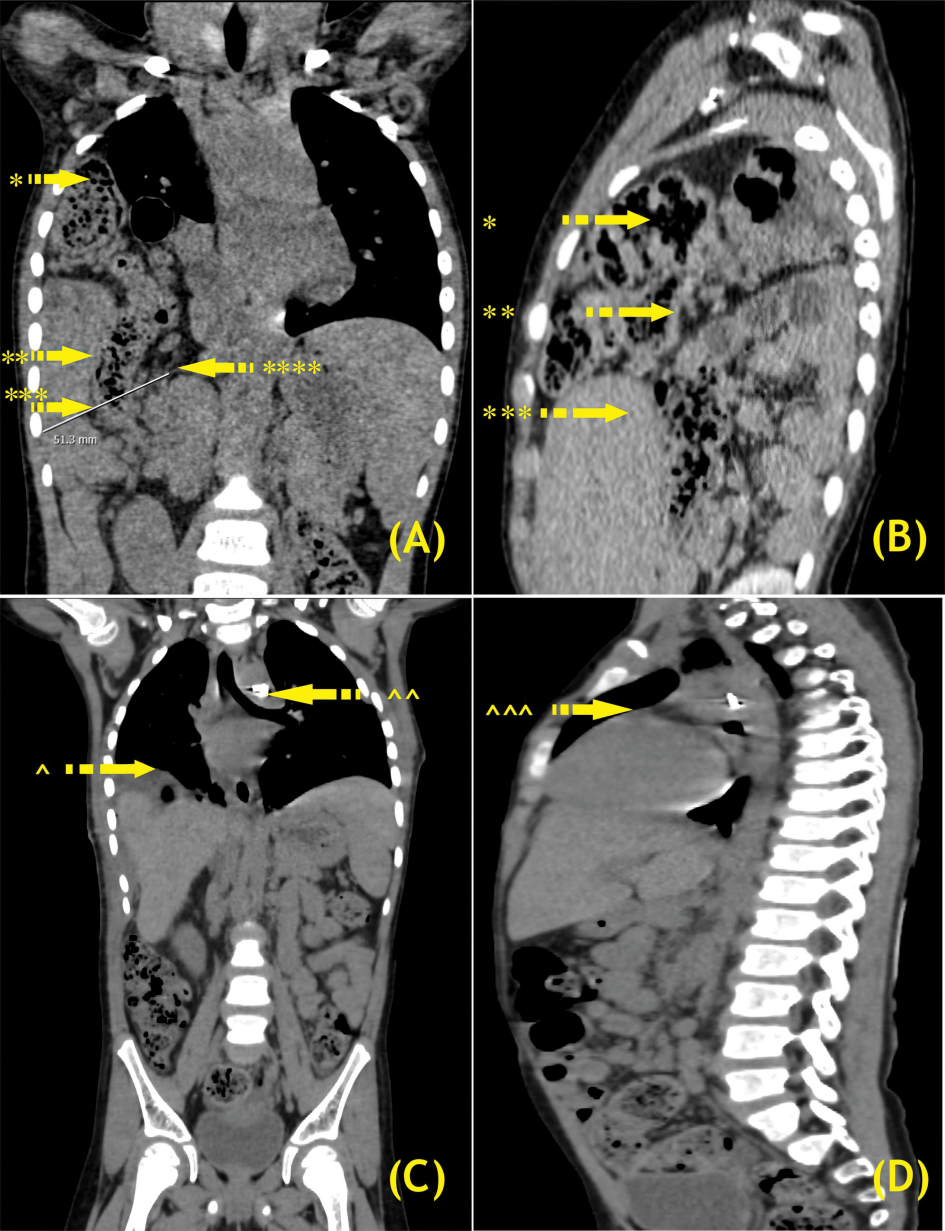
Chest and cardiac CT scans. (A) Preoperative coronal view showing right-sided BH with * herniating hepatic flexure, ** herniating liver segment VIII, *** defect along right posterolateral diaphragm, and **** crura of the right hemidiaphragm. (B) Preoperative sagittal view showing * right sided BH with *** herniation of liver segment VIII, and ** jejunal loops. (C) Postoperative coronal view displaying metallic artifacts from PDA ligation and ^ right CDH primary repair with Parietex™ Optimized Composite Mesh (Covidien). ^^ Postoperative metallic artifacts from PDA ligation. (D) Postoperative sagittal view showing postoperative metallic artifacts from PDA ligation (^^^). CT, computed tomography; BH, Bochdalek hernia; PDA, patent ductus arteriosus; CDH, congenital diaphragmatic hernia. (Image credits: Dr. Viral B. Patel and Dr. Jaimin P. Trasadiya)

Urinalysis and electrocardiogram results were within the reference ranges. A high-resolution CT scan of the lungs and cardiac CT revealed a Bochdalek hernia with a defect of approximately 5 cm in the right posterolateral diaphragm. The hernia contained the hepatic flexure of the colon, jejunal loops, and segment VIII of the liver, which were herniated into the right thoracic cavity, compressing the right lung and causing complete collapse of the right lower lobe. A 4.1-mm tubular channel connected the distal aortic arch to the undersurface of the main pulmonary artery, indicating the presence of a PDA (Figure 2).

After obtaining written informed consent from the patient's parents, surgery was planned to treat both the right-sided diaphragmatic hernia and the PDA.

Anesthesia and pain management

Surgery was performed under general anesthesia using a cuffed endotracheal tube (size 4.5 Fr) with an epidural catheter for postoperative pain management. The epidural catheter was placed at the T7-T8 intervertebral space. A test dose of 0.5 mL of 0.1% lignocaine with adrenaline was administered, followed by a bolus of 6 mL of 0.25% levobupivacaine with 25 mcg fentanyl. Continuous infusion was maintained at 4 mL/hr with 0.25% levobupivacaine and fentanyl (1 mcg/mL). Starting on postoperative day 1, fentanyl was discontinued, and levobupivacaine was tapered until postoperative day 3.

Surgical procedure

**Figure 3:**
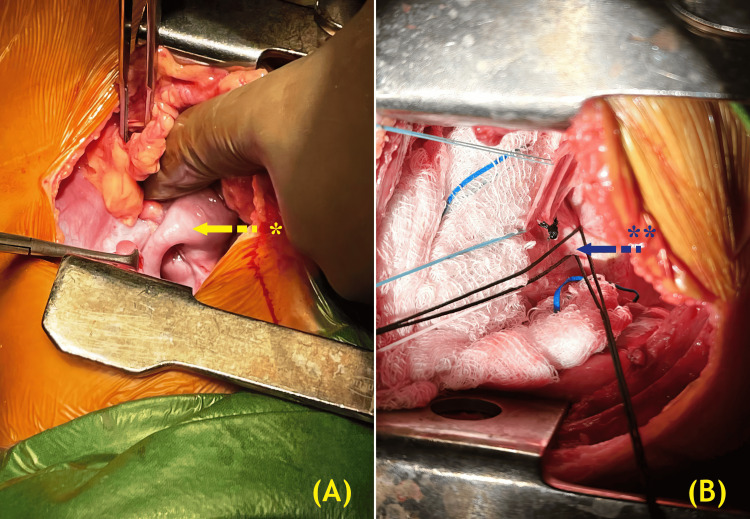
Right thoracotomy exposure showing (A) hernia contents, including the ileocecal appendix, cecum, ascending colon, and distal ileal loops. The yellow arrow and * indicate the appendix and small bowel in the right hemithorax. (B) PDA, looped before ligation during left thoracotomy exposure. The blue arrow and ** mark the PDA looped prior to ligation. PDA, patent ductus arteriosus. (Image credits: Dr. Vishal V. Bhende)

Two incisions were made for the surgical procedure: a left posterolateral thoracotomy for the PDA and a right posterolateral thoracotomy for the CDH repair (Figure 3).

**Figure 4:**
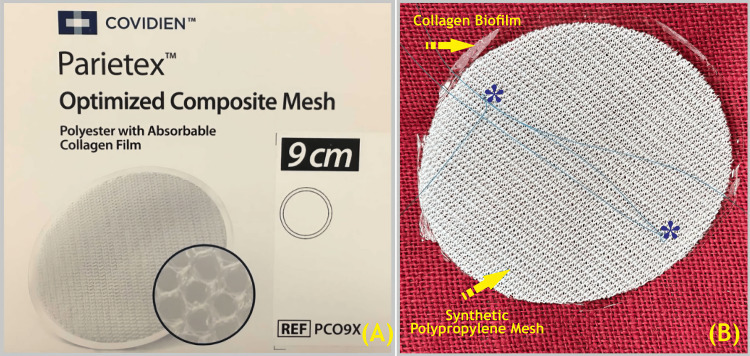
Covidien Parietex™ Optimized Composite Mesh (PCO9x). (A) box display and (B) show the mesh with * polypropylene suture haptics for mesh fixation. Covidien Parietex™ Optimized Composite Mesh 9 cm © 2024 by Covidien is licensed under CC BY-NC-SA 4.0 . Permitted and Attributed by Covidien Parietex™ Optimized Composite (PCOx) Mesh 9 cm © 2024 Image source: https://images.app.goo.gl/Kyxw2x27HygjauZq8 under a Creative Commons license (https://creativecommons.org/licenses/by-nc-sa/4.0/) (Image credits: Dr. Vishal V. Bhende)

Intraoperative findings included liver eventration into the right thoracic cavity, bowel loops, and the appendix. Adhesions between the diaphragm and the lower part of the lung were identified and released, after which the hernial sac was removed. The diaphragmatic defect was repaired with interrupted 2-0 polydioxanone sutures and reinforced with a 9-cm Parietex™ Optimized Composite Mesh (Covidien, Dublin, Ireland; Figure 4).

Following the repair, the right lung expanded normally. An intercostal chest drain was placed in the right sixth intercostal space and the thoracotomy incision was closed. A similar procedure was performed on the left side for PDA ligation with metallic clips, including chest tube placement, and the thoracotomy was closed.

Results

Postoperatively, the patient had no cough, sputum production, or dyspnea symptoms. She remained in the cardiac surgical intensive care unit for four days and was discharged after a total hospital stay of nine days. A chest X-ray and a follow-up CT scan of the chest, abdomen, and pelvis before discharge showed normal findings. The child is on regular follow-up and remains asymptomatic with no recurrence.

## Discussion

The association between CDH and congenital heart defects (CHD) is uncommon but well-documented. Approximately 15% of infants with CDH are found to have concurrent CHD, a prevalence that rises to 28% when stillborn infants are included, indicating a significant link between CDH, CHD prevalence, and survival rates [18]. Infants with both CDH and CHD have lower survival rates (52%) than those with isolated CDH (73%), and those with critical CHD have even lower survival (32%) [19].

The foramen of Bochdalek, a 2-cm to 3-cm opening in the posterolateral diaphragm during fetal development, typically closes by the eighth week as the pleuroperitoneal membranes fuse with the septum transversum [7,11]. Incomplete fusion can result in a hernia, first described by Bochdalek in 1848, according to Kumar et al. and Alam and Chander [8,20]. In infants, BH often presents with severe respiratory distress and cyanosis, requiring urgent surgery [8]. In adults, BH may be asymptomatic and incidentally discovered [8,11], although symptomatic presentations can include chest pain, respiratory distress, abdominal pain, nausea, vomiting, and complications such as intestinal incarceration or gastrointestinal perforation [7,10,12,14,20]. Symptoms may be intermittent if the herniated viscera spontaneously return to the abdomen [12].

Left-sided BHs are more common, occurring in 85% of cases, as the left diaphragm closes later during development. Herniated organs often include the stomach, ileum, colon, and spleen, while right-sided hernias may also involve the liver, kidney, and intestines [13]. Common symptoms include thoracic and abdominal pain, as seen in our patient, as well as occasional respiratory distress or bowel obstruction [7]. CT scanning is highly accurate for diagnosing BH, and its expanded use has led to more incidental findings of asymptomatic cases [7,12,14,20]. Ultrasonography is useful for prenatal detection of CDH, while magnetic resonance imaging can help assess thoracic masses relative to the diaphragm [20]. In this case, the diagnosis was initially suggested by chest X-ray and confirmed by CT. Surgery remains the preferred treatment, with a mortality rate under 4% for elective procedures and 32% for emergencies [7,8]. For our patient, the CDH and CHD were addressed via two separate surgical approaches [21]. Primary repair with reinforcement using prosthetic materials - such as biologic, composite, or synthetic mesh - offers a reliable solution (Table 1) [22-26].

**Table 1:** Various prosthetic meshes used for repair of CDH and traumatic diaphragmatic rupture CDH, congenital diaphragmatic hernia; HADM, human acellular dermal matrix; SIS, small intestine submucosa.

Author and year	Study design	Mesh material	Type of mesh
Teicher et al., 2010 [22]	Case report	HADM	Biologic
Pulido et al., 2011 [23]	Case report	HADM	Biologic
Al-Nouri et al., 2012 [24]	Case series	HADM/SIS	Biologic
Xin Yuan et al., 2020 [25]	Case report	Parietex ^TM ^optimized composite (polyester with absorbable collagen film and preplaced sutures)	Composite
Emrah Aydın et al., 2020 [26]	Original article	GORE-TEX^R^ or DUALMESH^R^ (W.L. Gore & Associates, Flagstaff, Arizona, USA)	Synthetic

Approximately 10% to 38% of BH cases include a hernial sac, though most do not [8,12]. Surgical approaches for BH repair vary, including thoracoscopy, laparoscopy, thoracotomy, and laparotomy. Some experts recommend thoracotomy due to the caution needed in dissecting adhesions between thoracic viscera and the hernial sac, while others advocate for laparotomy, which allows for better evaluation and management of potential malrotation, visceral incarceration, or obstruction [8,27,28]. For right-sided BHs, where liver herniation is common, a combination of laparotomy and thoracotomy may be advantageous [8,29]. Minimally invasive techniques are generally recommended for adult BH repairs [8,30,31].

In our case, the hernia was right-sided and involved the ileocecal appendix. A 2009 review of BH cases in children and adults identified the colon in one-third of cases, with only 11 right-sided hernias and no cases involving the ileocecal appendix [8]. Costa Almeida et al. described the first adult BH case with an ileocecal appendix and reviewed 25 other adult cases involving the colon, none of which included appendix herniation [32]. They referred to their case as the “Almeida-Reiss hernia,” noting it as the first adult case of right-sided BH involving the ileocecal appendix (Table 2) [8-11, 14, 20, 32-51].

**Table 2:** Case reports of right-sided BH with hernia contents ^a^All cases had no ileo-cecal appendix BH, Bochdalek hernia; NS, age not specified; C, colon; TC, transverse colon; RC, right colon; LC, left colon; SB, small bowel; St, stomach; Om, Omentum; HepF, hepatic flexure of the colon; RL, right lobe of the liver; Ce, cecum; Gb, gall bladder; RO, right ovary; RK, right kidney.

Author and year	Number of cases	Age in years	Side	Content^a^
Kumar et al., 2009 [8]	1	46	Left	TC
Terzi et al.,2008 [9]	1	70	Right	C, Om
Kavanagh et al., 2008 [10]	1	76	Right	TC
Granier et al., 2010 [11]	1	54	Right	Ileum, Ce, RC
Slesser et al., 2011 [14]	1	37	Right	RC
Wg Cdr A Alam et al., 2005 [20]	1	35	Right	St, SB, Colon, RL
Losanoff et al., 2004 [33]	1	29	Left	TC, Om
Esmer et al., 2008 [34]	1	42	Left	LC
Rout et al., 2008 [35]	1	35	Right	TC (blind loop)
Laaksonen et al., 2009 [36]	1	38	Right	HepF, RL
Kocakusak et al., 2005 [37]	1	21	Left	TC
Chai et al., 2005 [38]	1	46	Left	C
Hamoudi et al., 2004 [39]	1	41	Left	St, TC, Spleen
Court et al., 2003 [40]	1	40	Right	C, Liver
Kanazawa et al., 2002 [41]	1	63	Right	TC
Harinath et al., 2002 [42]	1	NS, Adult	Left	St, C
Iiai et al., 1997 [43]	1	38	Left	TC
Ohura et al., 1996 [44]	1	35	Left	SB, C
Kashima et al., 1993 [45]	1	70	Left	C
Sinha et al., 1989 [46]	1	70	Right	RC, TC
Rimpiläinen et al., 2002 [47]	1	22	Right	Liver, Gb, RC, Ileum, RO
Gymovsky and Schifrin 1983 [48]	1	20	Right	C
Fraser et al., 2009 [49]	1	75	Right	C, SB, RK
Zenda et al., 2000 [50]	1	69	Right	Ileum, TC, Gb
Mohammadhosseini and Shirani 2008 [51]	1	NS, Adult	Left	C

Through a literature review, we found one report of a pediatric patient with right-sided BH and an inflamed appendix in the thoracic cavity but without concurrent CHD [52]. This makes our case unique as the first reported and surgically treated instance of a congenital right-sided BH with herniation of the ileocecal appendix into the thoracic cavity and concurrent CHD.

## Conclusions

We presented a unique case of a four-year-old pediatric patient with a right-sided congenital BH involving the ileocecal appendix and concurrent CHD, specifically PDA. This rare presentation was identified through clinical evaluation and imaging, followed by successful surgical intervention. The rarity of this condition and its nonspecific symptoms often lead to delayed diagnosis or misdiagnosis. A high index of clinical suspicion is essential for timely diagnosis and management. As the first reported case of a pediatric right-sided BH with CHD (PDA) involving the ileocecal appendix, we propose naming this entity the Bhende-Pathak hernia, representing a pediatric variant of BH.
